# Toxicity of Ablative Radiation Therapy in the Management of Patients with Child-Pugh B/C Liver Function and Unresectable Hepatocellular Carcinoma (HCC)

**DOI:** 10.3390/cancers18040681

**Published:** 2026-02-19

**Authors:** William Sperduto, Taofik Oyekunle, Donna Niedzwiecki, Christine E. Eyler, Brian Czito, Christopher G. Willett, Devon Godfrey, Joseph K. Salama, Manisha Palta, Sarah J. Stephens

**Affiliations:** 1 Department of Radiation Oncology, Mayo Clinic, Phoenix, AZ 85054, USA; sperduto.william@mayo.edu; 2 Department of Biostatistics and Bioinformatics, Duke University Medical Center, Durham, NC 27710, USA; 3 Department of Radiation Oncology, Duke University Medical Center, Durham, NC 27710, USA; christine.eyler@duke.edu (C.E.E.); brian.czito@duke.edu (B.C.); christopher.willett@duke.edu (C.G.W.); devon.godfrey@duke.edu (D.G.); joseph.salama@duke.edu (J.K.S.); manisha.palta@duke.edu (M.P.); 4 Department of Radiation Oncology, Durham Veterans Affairs Medical Center, Durham, NC 27705, USA

**Keywords:** hepatocellular carcinoma, HCC, stereotactic body radiotherapy, SBRT, Child-Pugh, radiation-induced liver dysfunction, RILD

## Abstract

Background/Objectives: Hypofractionated ablative radiation is an increasingly popular option for patients with hepatocellular carcinoma (HCC). However, concern remains about the risk for radiation-induced liver toxicity in patients with decompensated liver function. Methods: We retrospectively identified patients with underlying Child-Pugh (CP) B or C liver function treated at our University and Veterans Affairs (VA) departments from 2014 to 2019. Primary endpoints included treatment-related toxicity and dosimetric parameters. Results: 38 patients were included in the analysis. Most patients (98%) had CP B or Albumin-Bilirubin (ALBI) grade 2–3 (100%) liver disease. The median dose was 50 Gy (range 30–50) delivered in 5 or 10 fractions. Most patients had a single tumor treated (66%) with a median size of 3.1 cm (Interquartile Range (IQR) 2.3–4.1). The mean liver dose was 9.28 Gy (IQR 6.76–13.64) with a liver D800cc of 3.99 Gy (IQR 1.41–8.02). All patients completed their intended course with a median follow-up of 43 months. Four patients (10.3%) developed non-classical radiation-induced liver disease (RILD), comparable to the rate for patients with CP A function treated contemporaneously (8.3%). Otherwise, one patient (2.6%) experienced acute grade 3+ (non-RILD) hepatobiliary toxicity, while one patient (2.6%) experienced late grade 3+ hepatobiliary toxicity. Local control was promising with 2-year freedom from progression *in the treated lesion* of 73% (95% CI 38–91%). Median overall survival was 12 months (95% CI 5–25 months). Conclusions: Ablative radiation for patients with decompensated liver function and HCC appears well tolerated with low rates of RILD and encouraging local control. With careful selection, these patients should be considered for inclusion in future randomized trials.

## Simple Summary

Hepatocellular carcinoma (or primary liver cancer) remains a leading cause of cancer-related deaths, with incidence rates increasing in certain parts of the world. Treatment options outside of surgical resection or transplant remain poorly defined, particularly for those with decompensated underlying liver function, leaving few available treatment options. The role for ablative radiation therapy for these patients has been limited historically given understandable concern regarding the potential for radiation-induced liver dysfunction. Our series evaluating the use of ablative radiation therapy in those with decompensated liver function (primarily Child-Pugh B) demonstrated that treatment is well tolerated with low rates of radiation-induced liver dysfunction and encouraging rates of local tumor control.

## 1. Introduction

Primary liver cancer (hepatocellular carcinoma or HCC) is the second-leading cause of cancer death in men and the sixth-leading cause in women worldwide, with incidence rates increasing in certain parts of the world, including Western Europe and North America [1]. Chronic hepatitis B and C infections, heavy alcohol consumption, obesity, type II diabetes, and tobacco use are the main determinants contributing to this increasing disease burden. While five-year relative survival has improved from 3 to 18% over the last forty years, the five-year overall survival for patients with localized disease remains only 33%. The management of HCC outside of surgical resection and/or transplant remains poorly defined; therefore, evaluating alternative local treatment options is necessary as a bridge to transplant or for those patients who are not transplant candidates [2].

Ablative hypofractionated radiation therapy is an evolving, increasingly attractive non-invasive option for patients with unresectable, non-metastatic HCC [3]. However, the role for this therapy for patients with decompensated liver function (CP B or C) remains poorly defined, given the understandable concern for radiation-induced liver disease (RILD). The utilization of hypofractionated radiation for patients in this population is also a balance of competing risks consisting of worsening underlying liver dysfunction (tumor- and/or cirrhosis-induced), medical comorbidities, and death from other causes. Available data evaluating the use of Stereotactic Body Radiotherapy (SBRT) or hypofractionated radiation therapy in the management of patients with moderately decompensated liver function remains limited, with only two small prospective series examining SBRT for HCC in patients with CP B and C disease [4,5]. While both were limited by small sample size, they suggest patients with moderate liver impairment may benefit from SBRT with acceptable treatment-related toxicity. Additionally, the optimal dose and fractionation for patients with impaired liver function remain unknown. The purpose of the current study was to retrospectively evaluate treatment-related toxicity of patients with underlying CP B or C liver function undergoing SBRT or hypofractionated radiation therapy at our University and affiliated Veterans Affairs (VA) hospitals. We also evaluated rates of local control in the treated tumor, overall survival, and dosimetric parameters for those patients treated at our institution.

## 2. Materials and Methods

Study design: We retrospectively identified all patients with unresectable, non-metastatic HCC and underlying CP B or C liver function who were treated with SBRT or hypofractionated radiation therapy (HIGRT or 6–10 fractions) at our University and Veterans Affairs (VA) radiation oncology departments from 2014 to 2019.

Appropriate patients were identified during multidisciplinary tumor boards following interdisciplinary discussion of the patient’s clinical history and appropriate treatment options. Both our University and VA hospitals serve as referral centers for a large geographic region. Patients were immobilized supine utilizing a customized immobilization device (e.g., Vac-Lok^TM^ (CIVCO Medical Solutions, Orange City, IA, USA) or similar device). Respiratory motion was assessed, and appropriate respiratory motion management was utilized as necessary per the treating physician’s discretion (including the use of breath hold, abdominal compression, or Active Breathing Coordinator (ABC)). To allow for better target delineation, both of our affiliated departments utilize bolus-tracked triphasic scans for Computed Tomography (CT) simulation as previously described [6]. Diagnostic imaging (triphasic CT, Magnetic Resonance Imaging (MRI), and/or Positron Emission Tomography (PET) scans) was fused for better target delineation and treatment planning as available. Treatment was delivered utilizing either an intensity-modulated radiation therapy (IMRT) or volumetric modulated arc therapy (VMAT) technique. Dose and fractionation were chosen at the discretion of the treating radiation oncologist based on the size of the treated tumor, tumor location, the patient’s underlying liver function, and doses to surrounding organs at risk (OAR). Dose constraints were also at the discretion of the treating physician; however, the following represent typical constraints utilized within our institution [Table 1].

Statistical analysis: Primary endpoints included treatment-related toxicity and evaluation of dosimetric parameters for the liver and nearby organs at risk (OARs). Acute toxicity was defined as that occurring ≤90 days from the completion of treatment versus late toxicity occurring >90 days from treatment completion. Low-grade toxicity was defined as grades 1–2 (Common Terminology Criteria for Adverse Events, CTCAE v5.0), and high-grade toxicity was defined as grade 3 or higher. Non-classical radiation-induced liver disease (RILD) was defined as an increase of 2 or more points in CP score from pre-treatment baseline within 90 days of treatment completion. Secondary endpoints included freedom from progression in the treated tumor (measured from treatment completion date until documented progression of disease within the treated lesion) and overall survival (measured from treatment completion date until death from any cause).

Patient, disease, and treatment characteristics were stratified by treatment site (University versus VA Hospital), summarized, and compared using chi-square tests for categorical variables and Wilcoxon rank-sum or Kruskal–Wallis tests for continuous variables. Kaplan-Meier curves were used to estimate the probability of local control (or freedom from progression in the treated lesion) and overall survival.

## 3. Results

Thirty-eight (38) patients with HCC and underlying CP B or C liver function received SBRT or HIGRT at our University Hospital or affiliated Veterans Affairs Medical Center between 2014 and 2019 with a median follow-up of 43 months. Most patients were male (87%) with a median age at diagnosis of 65 (IQR 61–72). The entire VA cohort was male, likely given the historical preponderance of male veterans in the U.S. Armed Forces. The most common etiologies for cirrhosis included chronic viral hepatitis (39%), concomitant alcohol use and viral hepatitis (27%), heavy alcohol use (16%), and metabolic dysfunction-associated steatotic liver disease (MASLD, previously known as nonalcoholic fatty liver disease or NAFLD) (11%). Most patients (98%) had CP B liver function prior to radiation therapy (B7 62%, B8 21%, B9 15%), with only a single patient having CP C liver function (C10). Alternatively, 69% of patients had ALBI Grade 2 pre-treatment liver function [Table 2].

Median treated lesion size was 3.1 cm (IQR range 2.3–4.1) with a median gross tumor volume (GTV) of 34.7 cc (IQR range 15–121.92) and planning target volume (PTV) of 86.17 cc (IQR range 44.92–223.18). Treatment intent was most often definitive therapy (62%), with fewer patients treated for salvage (26%) or bridge to transplant (8%). Most patients (66%) were treated for a single tumor with a median delivered dose of 50 Gy (range 30–50) given in 5–10 fractions. All patients were treated with the IMRT or VMAT technique, most (93%) with some form of respiratory motion management. Mean liver dose (MLD) was 9.28 Gy (IQR 6.76–13.64), and the D800cc to the liver was 3.99 Gy (IQR 1.41–8.02). Additional disease and treatment-related characteristics are summarized in Table 2 and Table 3.

**Figure 1:**
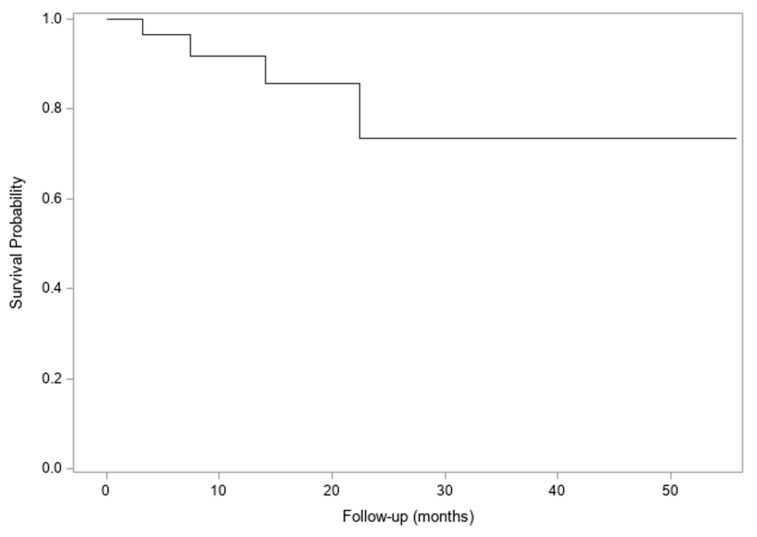
Freedom from progression in treated lesion. Median time to progression in the treated lesion [months] was not reached. Two-year freedom from local progression was 73% (95% CI 38–91%).

**Figure 2:**
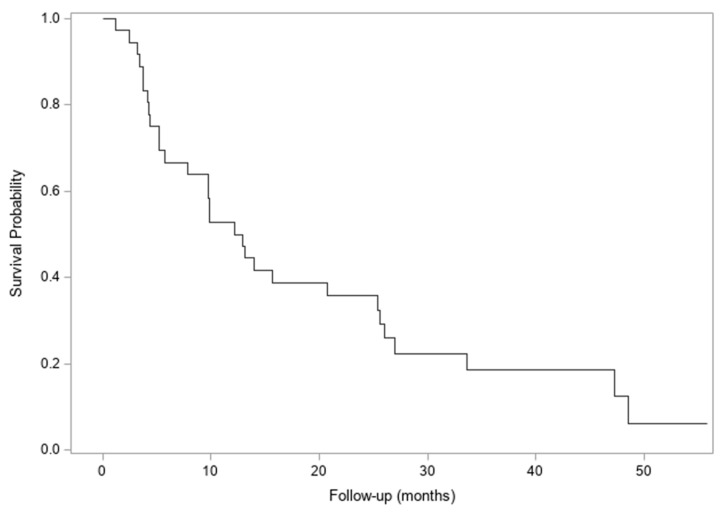
Overall survival. Median overall survival was 12 months (95% CI 5–25).

Rates of local control (as measured by the largest dimension of the treated lesion compared to baseline) were high, with a 2-year freedom from local progression of the treated lesion of 73% (95% Confidence Interval (CI) 38–91%) [Figure 1]. Median time to progression of the treated lesion was not reached, and median overall survival was 12 months (95% CI 5–25 months) [Figure 2].

One patient (2.6%) experienced acute grade 3+ (non-RILD) hepatobiliary toxicity (transient transaminitis, baseline CP B7). Four patients (10.3%) experienced non-classical radiation-induced liver disease (RILD) following treatment (3 with baseline CP B7; 1 with baseline CP B8), as defined by an increase in CP score of 2 or more points within 90 days of treatment, compared to 8.3% for patients with CP A liver function treated during a similar period at our institution [7]. Additionally, one patient (2.6%) experienced late grade 3+ hepatobiliary toxicity (stenosis, baseline CP B7).

## 4. Discussion

There are an increasing number of non-surgical, local therapy options for those with unresectable HCC, including radiofrequency ablation (RFA), microwave ablation (MWA), transcatheter arterial chemoembolization (TACE), transarterial radioembolization with Yttrium-90 (TARE-Y90), and ablative external beam radiation therapy. Ongoing studies comparing outcomes should help to clarify appropriate treatment selection in a multidisciplinary fashion. For ablative radiation therapy, concerns remain about potential liver toxicity, especially in patients with decompensated liver function (CP B/C). Therefore, we conducted one of the largest analyses to date of HCC patients with decompensated liver function treated with ablative radiation. We found that contrary to concerns, in our experience, ablative radiation can be delivered safely with low toxicity and RILD rates, comparable to patients with more mild liver impairment (CP A). There are also ongoing efforts to further delineate differential radiotherapy constraints based upon the patient’s underlying liver function [Supplemental Table S1]. Additionally, we found that treated tumor control remained high in these patients. However, opportunities to improve overall patient outcomes remain, as evidenced by the difference between local control and overall survival in our cohort, highlighting the ongoing impact of competing risk(s). Of note, our analysis did not identify enough treated patients with CP C liver function to make any meaningful conclusions for this patient population. We have begun an institutional study to address this scarcity of data.

*Radiation-Induced Liver Toxicity (RILD):* Two types of RILD have been described, including classical and non-classical disease. Classical RILD involves central vein occlusion and obliteration, retrograde congestion, and hepatocyte necrosis, whereas non-classical RILD is associated with hepatocellular loss/dysfunction and sinusoidal endothelial damage [8]. Specifically, four patients (10.3%) in the current study experienced non-classical RILD following radiation as defined by an increase in CP score of 2 or more points within the first 90 days following treatment (with the timeframe helping to distinguish RILD from natural progression of underlying liver dysfunction, but the potential for overlap remains). This is in comparison to the 63% at 90 days [4] and 17% at 6 months [5] seen in several previously published series. These data are important, as some have been hesitant to adopt ablative radiation as a therapeutic strategy for HCC patients given the perceived risk for RILD. Many of those who have adopted SBRT for HCC in patients with compensated function have avoided it in those patients with relatively decompensated function. Our data suggest that ablative radiation can be safely used with appropriate caution in patients with CP B7, B8, and B9 function.

By studying the safety and efficacy of SBRT in early-stage HCC patients with CP B7-C10 disease, Lee et al. found change in CP after SBRT to be prognostic, while baseline scores were not, suggesting advanced CP scores alone may not be sufficient to disqualify patients from receiving radiation therapy. The authors evaluated 23 patients with HCC and underlying CP B/C liver function who received SBRT or hypofractionated radiation therapy to 30–50 Gy in 4–6 fractions. The authors used a thoughtful CP-based approach to determine median liver dose with constraints of 8 Gy for CP B7/8 and 5 Gy for CP B9/C10 [5]. In the current analysis, we documented a mean liver dose (MLD) of 9.28 Gy (IQR 6.76–13.64) with still acceptably low rates of RILD. Although the series published to date are small, including our own, they strengthen the argument for enrolling carefully selected HCC patients with decompensated liver disease in randomized clinical trials. Several recent and ongoing trials should help further clarify the role of ablative radiation therapy in this patient population [9,10]. Recent and ongoing improvements in the accuracy of radiation planning and delivery, including real-time adaptive planning, may further refine the population of eligible patients and our ability to spare normal tissues (importantly, unaffected liver).

We found acute hepatobiliary toxicity to be infrequent in patients with decompensated liver function undergoing ablative radiation. Specifically, only a single patient in our series experienced acute high-grade, non-RILD hepatobiliary toxicity (transient transaminitis), comparable to the rate seen in contemporaneously treated patients with CP A function. This finding is important, as some have voiced concern about the ability to accurately target and deliver radiation therapy to these patients, given potential difficulty imaging those with cirrhosis, as well as potential patient difficulty tolerating lying supine or with respiratory motion management strategies. Additionally, low risk of toxicity makes ablative radiation an attractive treatment option for these patients who may have poor performance status and are not appropriate candidates for other local therapies (e.g., interventional radiology procedures). Addressing the underlying cause(s) of a patient’s cirrhosis may also serve to affect their outcomes [11].

*Characterization of Liver Function:* The laboratory parameters included in CP scoring are based on predetermined cutoffs. Categorizing these continuous variables may be misleading and can also lower statistical power. This, coupled with the subjective scoring of ascites and encephalopathy, warrants evaluation of more objective definitions of liver function that can differentiate radiation-induced effects from natural disease progression [5]. The Model for End-stage Liver Disease (MELD) score is also likely insufficient, as it is thought to be less reliable in patients with underlying malignancy, in part due to cancer-related cachexia. To objectively characterize liver function, Johnson et al. developed and externally validated the ALBI score, based on albumin and bilirubin levels. ALBI risk stratifies patients, highlights distinct prognostic subgroups within CP A, and avoids interobserver variation in scoring. These advantages are likely to be important in future clinical trials [12]. However, further evaluation is needed to assess how ALBI performs in relation to specific CP scores. In a retrospective cohort study of 594 patients treated with SBRT, the predictive power between the CP and ALBI scoring systems was similar for the CP A population, but additional validation of ALBI in the CP ≥B7 population is required [13]. While several studies have shown ALBI can predict survival and monitor liver toxicity, especially in patients with minimal liver dysfunction, Toesca et al. also found ALBI to be a more sensitive risk stratification tool to recognize liver function recovery [14]. Baseline ALBI also appears to be more discriminatory than CP in predicting overall survival and outperformed CP in predicting post-SBRT toxicity [15].

*Limitations*: The retrospective nature of our single-institution study makes it susceptible to convenience sampling and potential selection bias, which may limit the generalizability of our results. Underlying differences in baseline patient characteristics (in the absence of a randomized trial) can make it difficult to distinguish treatment-related toxicity from natural disease progression. Finally, the small sample size (particularly of patients with CP C disease) limits the overall analysis and precludes any subgroup analyses.

## 5. Conclusions

Ablative radiation therapy for patients with unresectable, non-metastatic HCC appears to be reasonably well tolerated in those with moderately decompensated liver function at baseline (CP B7-B9) with low rates of RILD and encouraging local control within the treated tumor. With careful selection, radiation therapy appears to be a reasonable treatment option in this patient population, and this approach warrants ongoing prospective evaluation. However, our analysis did not include enough patients with CP C10+ disease to draw meaningful conclusions about the role of ablative radiation in this population. The current analysis adds to the existing small body of similar, previously published series.

## Figures and Tables

**Table 1:** Typical organs at risk (OAR) constraints utilized at our institution for 5- and 10-fraction ablative liver treatment. ITV: Internal Target Volume.

**Organs at Risk (OAR) Dose Constraints, 5 Fraction Treatment**		
OAR	Dose (Gy)	Volume (absolute or %)
Stomach	35	<0.035 cc
	26.5	<5 cc
Bowel (large or small)	40	<0.035 cc
	28.5	<20 cc
Duodenum	30	<0.5 cc
	18.3	<5 cc
Skin	38.5	<0.035 cc
	36.5	<10 cc
Chest Wall	60.4	<0.035 cc
	30	<50 cc
Rib	57	<0.035 cc
	45	<5 cc
Liver—ITV	21	<[1-(700cc/Vtotal)]%, where Vtotal is the volume of Liver—ITV (cc)
Kidney Cortex	17.5	<[1-(200cc/Vtotal)]%, Where Vtotal is the volume of Kidney Cortex—ITV (cc)
**Organs at Risk (OAR) Dose Constraints, 10 Fraction Treatment**		
OAR	Dose (Gy)	Volume (absolute or %)
Stomach	50	<0.035 cc
Bowel (large or small)	50	<0.035 cc
Duodenum	40	<0.035 cc
Chest Wall	50	<60 cc
	40	<120 cc
	30	<250 cc
Liver—ITV	30	<[1-(700cc/Vtotal)]%, where Vtotal is the volume of Liver—ITV (cc)
Liver	13	Mean dose
Kidneys	16	<50%

**Table 2:** Patient and Disease Characteristics by Site. Most patients had a single lesion (66%) diagnosed primarily by imaging characteristics alone (79%). Most patients were male (87%) and white (66%). Most (97%) had Child-Pugh (CP) B7-B9 or ALBI Grade 2–3 liver disease prior to radiation therapy. A single patient had CP C10 disease. ECOG: Eastern Cooperative Oncology Group. LI-RADS: Liver Imaging Reporting and Data System. AST: Aspartate Aminotransferase. ALT: Alanine Transaminase. PT/INR: Prothrombin Time and International Normalized Ratio.

	University (*n* = 16)	VA Hospital (*n* = 22)	Total (*n* = 38)	*p*-Value
Median age at diagnosis (IQR)	68 (59, 75)	65 (61, 69)	65 (61, 72)	0.594 ^1^
Race				0.212 ^2^
White	12 (75%)	13 (59%)	25 (66%)	
Black	2 (13%)	8 (36%)	10 (26%)	
Other	2 (13%)	1 (5%)	3 (8%)	
Sex				0.005 ^2^
Male	11 (69%)	22 (100%)	33 (87%)	
Female	5 (31%)	0 (0%)	5 (13%)	
ECOG performance status				0.579 ^2^
0	0 (0%)	1 (5%)	1 (3%)	
1	7 (44%)	13 (59%)	20 (53%)	
2	8 (50%)	7 (32%)	15 (39%)	
3	1 (6%)	1 (5%)	2 (5%)	
History of another cancer diagnosis				0.435 ^2^
Yes	4 (25%)	6 (27%)	10 (27%)	
No	12 (75%)	16 (73%)	28 (74%)	
Smoking history				0.283 ^2^
Yes	10 (63%)	15 (68%)	25 (66%)	
No	6 (38%)	4 (18%)	10 (26%)	
Missing	0 (0%)	3 (14%)	3 (8%)	
Single or Multiple liver lesions				0.715 ^2^
Single	10 (63%)	15 (68%)	25 (66%)	
Multiple	6 (38%)	7 (32%)	13 (34%)	
Method of diagnosis				0.303 ^2^
Biopsy	4 (25%)	3 (14%)	7 (18%)	
Imaging (LI-RADS criteria)	11 (69%)	19 (86%)	30 (79%)	
Surgery	1 (6%)	0 (0%)	1 (3%)	
Presence of underlying cirrhosis				-
Yes	16 (100%)	22 (100%)	38 (100%)	
Receiving dialysis (at least twice weekly)				0.387 ^2^
Yes	0 (0%)	1 (5%)	1 (3%)	
No	16 (100%)	21 (95%)	37 (97%)	
Probable cause of cirrhosis				
Alcohol	3 (19%)	3 (14%)	6 (16%)	-
Viral Hepatitis	9 (56%)	6 (27%)	15 (39%)	
Unknown	1 (6%)	2 (9%)	3 (8%)	
Alcohol plus Viral Hepatitis	0 (0%)	10 (46%)	10 (27%)	
MASLD (formerly NAFLD)	3 (19%)	1 (5%)	4 (11%)	
Portal vein thrombosis				0.011 ^2^
Yes	4 (25%)	0 (0%)	4 (10%)	
No	12 (75%)	23 (100%)	35 (90%)	
Presence of nodal metastases				0.791 ^2^
Yes	1 (6%)	1 (4%)	2 (5%)	
No	15 (94%)	22 (96%)	37 (95%)	
Largest tumor dimension (cm)	3.35 (2.85, 4.45)	2.70 (1.70, 3.50)	3.10 (2.30, 4.10)	0.103 ^3^
Second largest tumor dimension (cm)	2.85 (2.25, 4.00)	2.30 (1.50, 3.00)	2.60 (2.00, 3.30)	0.061 ^3^
Baseline Alpha-fetoprotein (AFP)	50.60 (6.70, 1030.70)	12.20 (6.40, 73.80)	14.40 (6.55, 96.30)	0.178 ^3^
Baseline AST	62.00 (37.00, 91.00)	60.00 (38.00, 88.00)	60.50 (38.00, 88.00)	0.905 ^3^
Baseline ALT	32.50 (24.00, 54.00)	43.00 (30.00, 54.00)	40.00 (28.00, 54.00)	0.424 ^3^
Baseline Alkaline Phosphatase	126.50 (99.50, 174.50)	171.00 (108.00, 212.00)	159.00 (107.00, 195.00)	0.138 ^3^
Baseline Sodium	136.50 (134.50, 138.50)	138.00 (136.00, 140.00)	138.00 (135.00, 139.00)	0.111 ^3^
Baseline Creatinine	0.95 (0.75, 1.25)	1.00 (0.86, 1.34)	1.00 (0.80, 1.30)	0.345 ^3^
Baseline Platelet Count	96.50 k (63.50, 132.50)	86.00 k (69.00, 118.00)	93.00 k (68.00, 118.00)	0.564 ^3^
Baseline Total Bilirubin	1.80 (0.95, 2.65)	1.10 (0.70, 2.20)	1.30 (0.70, 2.60)	0.112 ^3^
Baseline Albumin	2.95 (2.75, 3.30)	2.80 (2.50, 3.10)	2.90 (2.70, 3.30)	0.359 ^3^
Baseline PT/INR	1.00 (1.00, 1.00)	1.20 (1.12, 1.30)	1.12 (1.00, 1.25)	<0.001 ^3^
Encephalopathy				0.960 ^2^
Yes	2 (13%)	3 (13%)	5 (13%)	
No	14 (88%)	20 (87%)	34 (87%)	
Baseline Child-Pugh (CP) Score				0.296 ^2^
B7	10 (63%)	14 (61%)	24 (62%)	
B8	5 (31%)	3 (13%)	8 (21%)	
B9	1 (6%)	5 (22%)	6 (15%)	
C10	0 (0%)	1 (4%)	1 (3%)	
Baseline ALBI Grade				0.957 ^2^
2	11 (69%)	16 (70%)	27 (69%)	
3	5 (31%)	7 (30%)	12 (31%)	

^1^ Wilcoxon rank sum test; ^2^ Chi-Square test; ^3^ Kruskal–Wallis.

**Table 3:** Radiation Treatment Course Characteristics by Site. Most patients (62%) were treated with definitive intent. The median delivered dose was 50 Gy given in 5–10 fractions. The mean liver dose (MLD) was 9.28 Gy with a liver D800cc of 3.99 Gy.

	University (*n* = 16)	VA Hospital (*n* = 23)	Total (*n* = 39)	*p*-Value
Prior liver-directed therapies				0.440 ^1^
Yes	5 (31%)	10 (43%)	15 (38%)	
No	11 (69%)	13 (57%)	24 (62%)	
Biliary stent present during radiation				-
No	16 (100%)	23 (100%)	39 (100%)	
Machine type used for treatment delivery	16 (100%)	23 (100%)	39 (100%)	-
Linac				
Intent of radiation therapy				0.120 ^1^
Definitive	9 (56%)	15 (65%)	24 (62%)	
Salvage	4 (25%)	6 (26%)	10 (26%)	
Bridge to transplant	3 (19%)	0 (0%)	3 (8%)	
Adjuvant	0 (0%)	2 (9%)	2 (5%)	
Prior radiation to the liver				0.398 ^1^
Yes	0 (0%)	1 (4%)	1 (3%)	
No	16 (100%)	22 (96%)	38 (97%)	
Treatment delivery technique				<0.001 ^1^
VMAT	6 (38%)	23 (100%)	29 (74%)	
IMRT	10 (63%)	0 (0%)	10 (26%)	
Respiratory motion management				0.002 ^1^
Free breathing	2 (13%)	1 (4%)	3 (8%)	
Activated Breathing Control (ABC)	0 (0%)	8 (35%)	8 (21%)	
Breath-hold	9 (56%)	2 (9%)	11 (28%)	
Abdominal compression	5 (31%)	12 (52%)	17 (44%)	
Median radiation dose (Gy)	50.00 (31.50, 50.00)	50.00 (50.00, 50.00)	50.00 (45.00, 50.00)	0.052 ^2^
Mean number of fractions	7.50 (5.00, 10.00)	10.00 (5.00, 10.00)	10.00 (5.00, 10.00)	0.895 ^2^
Median elapsed days during radiation	11.00 (6.50, 11.50)	12.00 (6.00, 14.00)	11.00 (6.00, 14.00)	0.351 ^2^
Median GTV (cc)	85.33 (33.08, 147.46)	19.10 (8.00, 48.90)	34.70 (15.00, 121.92)	0.013 ^2^
Median PTV (cc)	219.95 (82.35, 300.04)	56.70 (31.50, 116.80)	86.17 (44.92, 223.18)	0.005 ^2^
Median liver volume (cc)	1392.31 (1296.98, 1748.39)	1654.60 (1390.20, 2203.70)	1620.40 (1328.29, 1914.10)	0.098 ^2^
Liver volume—GTV (cc)	1332.76 (1256.55, 1658.58)	1635.50 (1388.00, 1998.90)	1558.30 (1279.75, 1828.70)	0.052 ^2^
Liver volume—PTV (cc)	1262.93 (1136.82, 1538.94)	1576.80 (1371.90, 1915.50)	1470.36 (1163.55, 1750.70)	0.028 ^2^
Median liver dose (Gy)	12.71 (9.25, 15.67)	7.30 (5.32, 10.01)	9.28 (6.76, 13.64)	0.008 ^2^
Median liver D800cc (Gy)	6.96 (2.30, 8.65)	3.06 (1.19, 6.87)	3.99 (1.41, 8.02)	0.153 ^2^
Median bowel D0.035cc (Gy)	20.73 (7.49, 30.14)	4.16 (2.62, 32.29)	15.69 (2.87, 30.93)	0.380 ^2^
Median bowel D0.5cc (Gy)	18.50 (6.66, 24.68)	3.76 (2.52, 27.64)	11.31 (2.66, 27.64)	0.393 ^2^
Median bowel D20cc (Gy)	5.55 (2.20, 8.71)	2.18 (0.93, 8.75)	2.95 (1.58, 8.72)	0.329 ^2^
Median duodenum D0.035cc (Gy)	22.29 (9.96, 33.80)	5.23 (0.81, 8.97)	6.57 (1.94, 22.29)	0.078 ^2^
Median duodenum D0.5cc (Gy)	19.41 (9.21, 33.50)	3.96 (0.74, 8.34)	5.07 (1.62, 17.75)	0.044 ^2^
Median duodenum D5cc (Gy)	8.89 (7.27, 33.10)	1.16 (0.55, 6.02)	2.38 (0.86, 7.27)	0.032 ^2^
Median stomach D0.035cc (Gy)	9.43 (5.21, 14.26)	5.77 (3.97, 8.25)	6.36 (4.89, 10.85)	0.301 ^2^
Median stomach D0.5cc (Gy)	8.32 (4.87, 12.97)	5.36 (3.63, 7.61)	5.85 (4.62, 10.30)	0.270 ^2^
Median stomach D5cc (Gy)	7.03 (4.23, 9.32)	4.53 (2.77, 6.26)	4.87 (3.72, 8.96)	0.178 ^2^
Post-radiation liver treatment				0.862 ^1^
Yes	6 (38%)	8 (35%)	14 (36%)	
No	10 (63%)	15 (65%)	25 (64%)	

^1^ Chi-Square; ^2^ Kruskal–Wallis.

## Data Availability

Research data are stored in an institutional repository. All data generated or analyzed during this study are included in this published article (and any Supplementary Information Files).

## Supplementary Materials

The following supporting information can be downloaded at: https://www.mdpi.com/article/10.3390/cancers18040681/s1, Table S1: Proposed differential dose constraints for patients with underlying CP B liver function.

## Abbreviations

The following abbreviations are used in this manuscript:

HCC	Hepatocellular Carcinoma
CP	Child-Pugh
IQR	Interquartile Range
VA	Veterans Affairs
ALBI	Albumin-Bilirubin
RILD	Radiation-Induced Liver Disease (or Dysfunction)
SBRT	Stereotactic Body Radiation Therapy
HIGRT	Hypofractionated Radiation Therapy (or Hypofractionated Image-Guided Radiation Therapy)
ABC	Active Breathing Coordinator
CT	Computed Tomography
MRI	Magnetic Resonance Imaging
PET	Positron Emission Tomography
IMRT	Intensity-Modulated Radiation Therapy
VMAT	Volumetric Modulated Arc Therapy
OAR	Organ At Risk
ITV	Internal Target Volume
ECOG	Eastern Cooperative Oncology Group
LI-RADS	Liver Imaging Reporting and Data System
AST	Aspartate Aminotransferase
ALT	Alanine Transaminase
PT/INR	Prothrombin Time and International Normalized Ratio
CI	Confidence Interval
CTCAE	Common Terminology Criteria for Adverse Events
MASLD	Metabolic Dysfunction-Associated Steatotic Liver Disease (previously known as NonAlcoholic Fatty Liver Disease or NAFLD)
GTV	Gross Tumor Volume
PTV	Planning Target Volume
MLD	Mean Liver Dose
MELD	Model for End-stage Liver Disease
RFA	Radiofrequency Ablation
MWA	Microwave Ablation
TACE	Transcatheter Arterial ChemoEmbolization
TARE-Y90	TransArterial RadioEmbolization Yttrium-90
