# Supplementary material for: Toxicity of Ablative Radiation Therapy in the Management of Patients with Child-Pugh B/C Liver Function and Unresectable Hepatocellular Carcinoma (HCC)
Source: Cancers (Basel). 2026 Feb 19;18(4):681. doi: 10.3390/cancers18040681 (PMC12939832; doi:10.3390/cancers18040681)
Supplement: Supplementary file 1 [file cancers-18-00681-s001.zip › cancers-4134585-supplementary.pdf]

Table S1. Proposed differential dose constraints for patients with underlying CP B liver function.

5 Fraction Regimen	Constraint
Mean liver dose (MLD)	≤ 8 Gy
D500cc	≤ 10 Gy
10 Fraction Regimen	Constraint
Mean liver dose (MLD)	≤ 12 Gy
V25Gy	≤ 15%
